# Atlantoaxial dislocation with associated type II odontoid fracture in adolescent with cervical spondylitis tuberculosis: A case report^☆^

**DOI:** 10.1016/j.ijscr.2023.107920

**Published:** 2023-02-09

**Authors:** Singkat Dohar Apul Lumban Tobing, Januar Chrisant Fladimir Makabori

**Affiliations:** Department of Orthopaedic and Traumatology, Dr. Cipto Mangunkusumo Hospital, Faculty of Medicine Universitas Indonesia, Jakarta 10430, Indonesia

**Keywords:** Atlantoaxial dislocation, Odontoid fracture, Cervical spondylitis tuberculosis, Autologous bone graft

## Abstract

**Introduction and importance:**

Atlantoaxial dislocation is a loss of joint stability between the C1 (atlas) and C2 (axis) spine and could be associated with type II odontoid fracture. In a few previous studies, atlantoaxial dislocation with odontoid fracture has been reported to be the complication of upper cervical spondylitis tuberculosis (TB).

**Case presentation:**

A 14-year-old girl came with sudden neck pain and difficulty moving her head that has worsened in the last 2 days. There was no motoric weakness in her limbs. However, tingling in both hands and feet was felt. X-ray examination showed atlantoaxial dislocation with odontoid fracture. Traction and immobilization using Garden-Well Tongs obtained the reduction of the atlantoaxial dislocation. Transarticular atlantoaxial fixation using cerclage wire and cannulated screw with an autologous graft from the iliac wing was performed through the posterior approach. A postoperative X-ray showed stable transarticular fixation with excellent screw placement.

**Clinical discussion:**

The application of Garden-Well tongs as a treatment for cervical spine injury has been documented in the previous study with a low rate of complications such as pin loosening, the asymmetrical position of the pin, and superficial infection. The reduction attempt did not significantly improve Atlantoaxial dislocation (ADI). Thus surgical treatment of atlantoaxial fixation using cannulated screw and c-wire with the application of an autologous bone graft is performed.

**Conclusion:**

Atlantoaxial dislocation with an odontoid fracture in cervical spondylitis TB is a rare spinal injury. The use of traction with surgical fixation is needed to reduce and immobilize atlantoaxial dislocation and odontoid fracture.

## Highlights

• Atlantoaxial dislocation with odontoid fracture has been reported to be the complication of upper cervical spondylitis TB.
• Atlantoaxial dislocation with odontoid fracture in cervical spondylitis TB is a rare spinal injury.
• The use of traction with surgical fixation is needed to reduce and immobilize atlantoaxial dislocation.

## 1 Introduction

Atlantoaxial dislocation is a loss of joint stability between the C1 (atlas) and C2 (axis) spine and could be associated with type II odontoid fracture [1]. Pathological process of the C1 and C2 spine could lead to odontoid fracture and atlantoaxial dislocation [2]. Atlantoaxial dislocation with odontoid fracture has been reported to be a complication of upper cervical spondylitis TB in a few previous studies. Upper cervical spondylitis tuberculosis is very rare, with an incidence of 0,3–1 % of all spondylitis TB cases [3]. Atlantoaxial dislocation with odontoid fracture could lead to severe morbidity and mortality in several cases [4]. This case report aims to describe the initial and definitive treatment of atlantoaxial dislocation with an associated odontoid fracture in an adolescent with cervical spondylitis tuberculosis. We have reported this case report according to SCARE criteria [5].

## 2 Case illustration

**Fig. 1:**
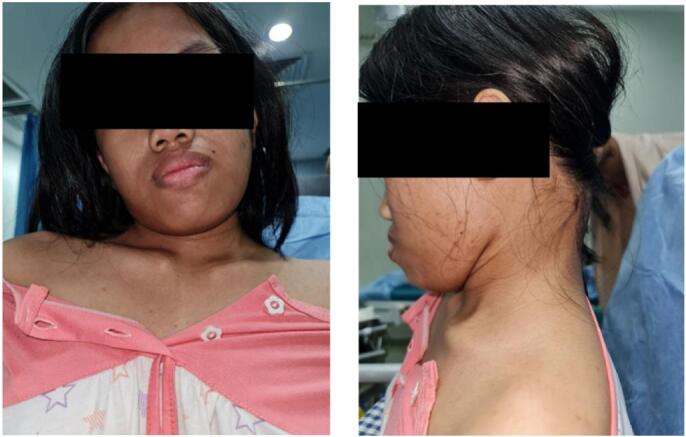
Physical examination with deformity in the C2 region.

**Fig. 2:**
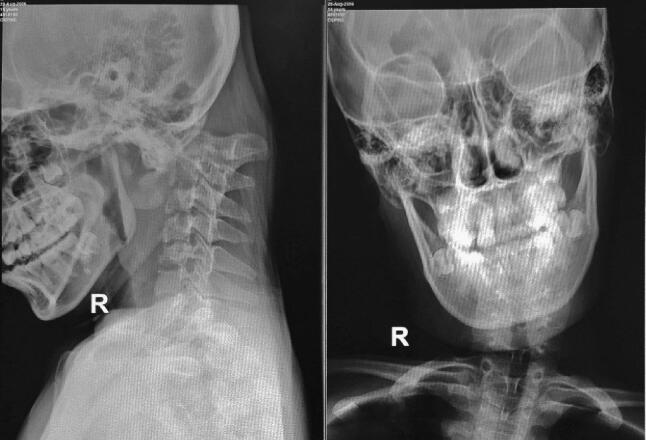
X-ray examination of the cervical spine showed atlantoaxial dislocation with type II odontoid fracture.

**Fig. 3:**
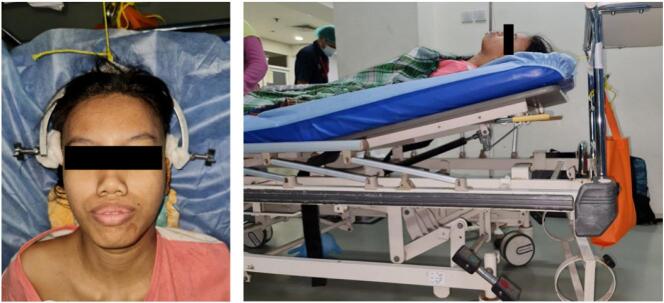
Immobilization using Garden-Well tongs.

**Fig. 4:**
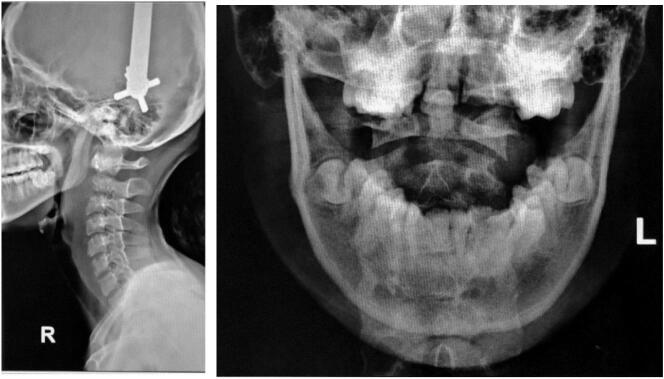
Post-traction radiographic evaluation.

**Fig. 5:**
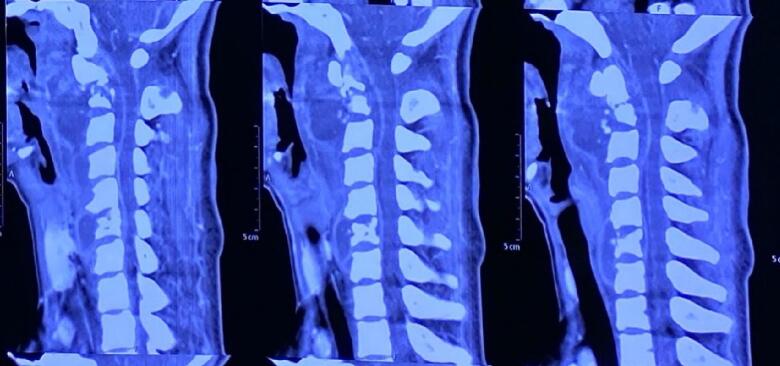
CT-scan of the cervical spine.

A 14-year-old girl came with sudden neck pain and difficulty moving her head that has worsened in the last 2 days. Previously, there was no history of cough, weight loss, fever over a long period, or suspicion of previous contact with TB. The patient felt a cracking sound on her neck before the sudden neck pain. There was no motoric weakness in her limbs. However, tingling in both hands and feet was felt. Previous neck pain was reported 3 months before the onset of acute pain. The local state of the neck showed deformity with a lump in the C2 region (Fig. 1, Fig. 2). The range of motion of the cervical spine was limited due to pain. The motor and sensory examination showed normal results despite urinary incontinence. Cervical X-ray examination in anteroposterior (AP) view, lateral, and open mouth, detailed with CT-scan, showed atlantoaxial dislocation with an odontoid fracture with atlantodental interval of 6.2 mm and space available for the cord (SAC) of 22.5 mm. Traction and immobilization using Garden-Well Tongs obtained the reduction of atlantoaxial dislocation (Fig. 3). Post-traction X-ray showed improvement in cervical spine alignment but no improvement in the ADI (Fig. 4). A computed tomography scan was then obtained, which confirmed type II odontoid fracture with the destruction of the C2 vertebral body and C6 vertebral body (Fig. 5). The patient was diagnosed with atlantoaxial dislocation with odontoid fracture type II due to suspected spondylitis tuberculosis infection.

**Fig. 6:**
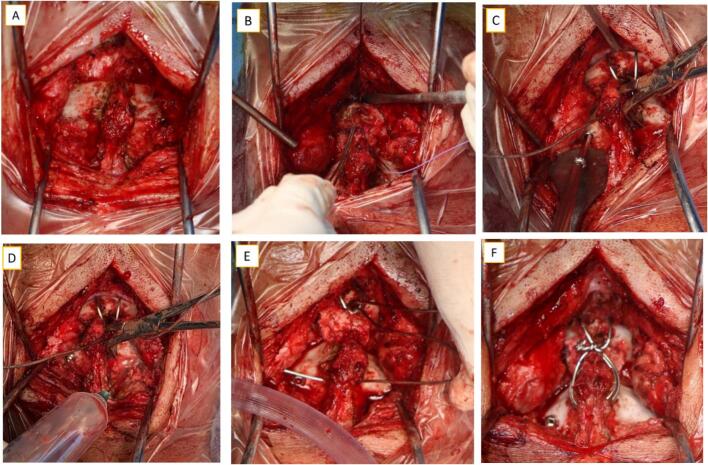
Intraoperative: A) cervical spine expose, B) C-wire insertion, C) cannulated screw insertion, D) biopsy and culture, E) bone graft application, F) final construct.

**Fig. 7:**
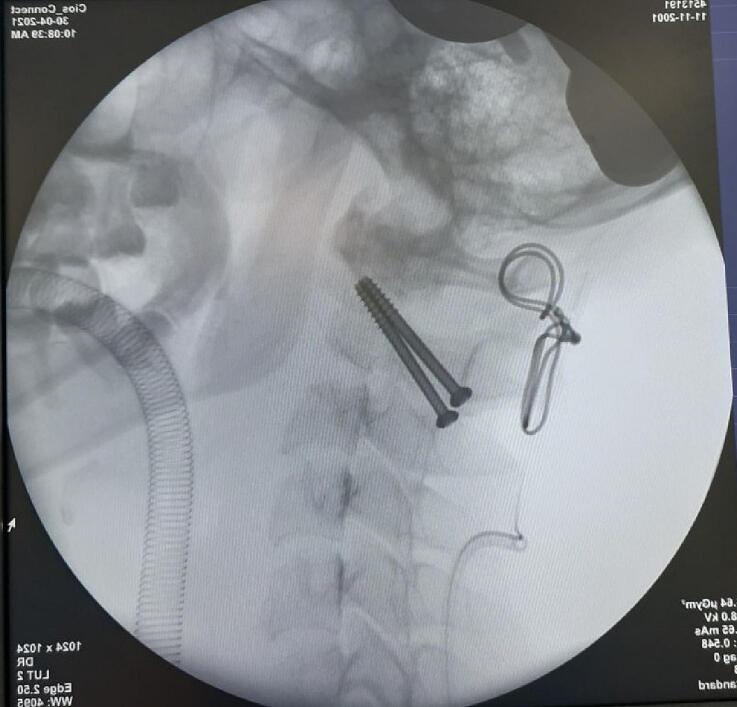
Postoperative X-ray cervical spine.

Transarticular atlantoaxial fixation using cerclage wire and a 3.5 mm cannulated screw (Synthes, Massachusetts. United States) with an autologous graft from the iliac wing was performed through a posterior approach (Fig. 6) with general anaesthesia and the patient was prepared in a prone position. During intra-operative, we also performed a biopsy and obtained tissue for culture. A postoperative X-ray showed stable transarticular fixation with excellent screw placement (Fig. 7). Post-operatively, the patient could mobilize typically without any neurological deficits, urinary incontinence was resolved, and anti-tuberculosis drugs were continued for up to 1 year post-operatively.

## 3 Discussion

Atlantoaxial dislocation with an odontoid fracture is a rare condition with fatal complications. The majority of patients have predisposing inflammatory conditions that lead to the instability of the joint [6]. The presentation of atlantoaxial dislocation with an odontoid fracture is neck pain and deformity, with or without neurological impairment. In this case, the 14-year-old female adolescent presented with neck pain and deformity without any neurological impairment. The patient felt a crack in the neck after a trivial trauma, which suggested the pathological nature of the cervical instability and fracture. The history of neck pain further suspected the prior pathological process several months before the fracture. Further CT-scan examination showed destruction of the odontoid process, C2 vertebral body and C6 vertebral bodies, which increased the suspicion of cervical spondylitis tuberculosis. Previous studies have reported atlantoaxial dislocation with the destruction of the odontoid process that leads to atlantoaxial instability [7]. The infectious process in the odontoid could lead to inflammation or infection spreading to the ligamentous structures, such as the transverse ligament and alar ligament, leading to atlantoaxial dislocation. Odontoid fracture in spondylitis TB has been described in other studies with the complications of retropharyngeal abscess and severe neurologic disorders [8]. In this case, the neurological deficit was not present and could be associated with large SAC in X-ray examination.

There was no previous systemic manifestation of prodromal TB infection in this patient. Systemic symptoms may be lacking, and early complaints may include neck discomfort or stiffness. Diagnosis may be delayed until advanced illness symptoms appear. Tuberculosis infection typically spreads retrogradely, reaching the craniovertebral joints. The infection destroys the bone and ligamentous components, resulting in cervicomedullary neural compression and occipitocervical or atlantoaxial instability [9]. This complex's disintegration can result in aberrant translational and rotational movements, resulting in severe morbidity and even death. The patient in this case presented with severe increasing neck pain, indicating instability [10], [11].

The treatment for atlantoaxial dislocation with odontoid fracture includes operative and non-operative modalities. Cervical traction is the most typical reduction technique to reduce atlantoaxial dislocation [6]. Moreau et al. have proposed a treatment pathway for atlantoaxial dislocation with the initial treatment attempt to reduce dislocation using a halo head brace or another traction device [12]. The use of cervical traction as a definitive treatment in atlantoaxial or atlantooccipital dislocation has been described in the study by Kumar et al. with no progressive neurological deficit [8]. Previous studies by Kumar et al. and Ould-Slimane et al. showed an immediate reduction with an improvement of the neurological deficit described in atlantoaxial dislocation with or without odontoid fracture [8], [13]. In this study, we applied Garden-Well tongs to reduce dislocation. The application of Garden-Well tongs as a treatment for cervical spine injury has been documented in a study by Saleh et al. with a low rate of complications such as pin loosening, the asymmetrical position of the pin, and superficial infection [14]. However, the lower pull-out strength of tongs and progressive decrease of the pull-out strength of tongs made halo traction more preferred. Previous study by Tian et al. reported skull traction with an initial weight of 3 kg followed by the gradual increase of weight until 12 kg in adults [15]. In this case, Garden-Well tongs reduction attempt did not show significant improvement of ADI, thus surgical treatment is required to stabilize the joint further [9].

Surgical treatment is then conducted with atlantoaxial fixation using cannulated screw and c-wire with the application of an autologous bone graft. The aim of surgical treatment has been described by Sinha et al. with anterior neural decompression, radical excision of epidural granulation, drainage of epidural abscess, removal of the infected bone, and biopsy [7]. Posterior arthrodesis was performed simultaneously after the anterior craniomedullary decompression [7]. Another study by Wang et al. has also proposed the use of both anterior and posterior approaches to achieve anterior debridement and decompression, and posterior stabilization [16].

Surgical stabilization could be conducted with transarticular screw fixation or occipitocervical fixation using a plate and screw [12], [17]. Transarticular fixation with cannulated screw stabilizes the atlantoaxial joint at the cost of restricting occipitocervical rotatory movement [6]. Occipitocervical fixation is an alternative to transarticular fixation if there is any technical limitation to transarticular fixation or destruction of the C1 vertebrae [7], [12]. Fixation of the odontoid fracture is no longer necessary as the joint is already stable [18]. The complications of occipitocervical fixation include vertebral artery injury, cerebrospinal leakage, wound dehiscence, wound infection, pneumonia, and death [6]. However, we did not observe any significant postoperative complications in this case. The application of iliac crest bone graft and sublaminar wire has been described in a previous study [7], [16]. The use of autologous bone graft in the atlantoaxial fusion has been described in the Sheehan et al. study to increase the rate of C1-C2 bone fusion [19]. A study by Yang et al. showed a significantly higher rate of bony fusion of the atlantoaxial joint, thus expressing the importance of autologous bone graft [20].

## 4 Conclusion

Although rare, atlantoaxial dislocation complications with odontoid fractures should always be considered in cases of TB upper cervical spondylitis. In this case, the use of traction with surgical fixation is essential to reduce and immobilize atlantoaxial dislocation.

## Consent

Written informed consent was obtained from the patient for publication of this case report and accompanying images. A copy of the written consent is available for review by the Editor-in-Chief of this journal on request.

## Provenance and peer review

Not commissioned, externally peer-reviewed.

## Ethical approval

Ethical approval was exempt/waived at our institution since all patient's identity is blinded throughout this manuscript.

## Sources of funding

The authors report no external source of funding during the writing of this article.

## Author contribution

SDALP: study concept or design, data collection, analysis and interpretation, oversight and leadership responsibility for the research activity planning and execution, including mentorship external to the core team.

JCFM: study concept or design, data collection and writing the paper.

## Guarantor

JCFM, MD.

## Research registration (for case reports detailing a new surgical technique or new equipment/technology)

Does not need any registration.

## Declaration of competing interest

We declare that all authors have no conflict of interest to declare.
